# Endovascular treatment of basilar tip aneurysms in the era of endosaccular flow disruption: a comparative study

**DOI:** 10.1007/s00234-020-02555-0

**Published:** 2020-09-23

**Authors:** Yigit Ozpeynirci, Bettina Hutschenreuter, Robert Forbrig, Hartmut Brückmann, Thomas Liebig, Franziska Dorn

**Affiliations:** Department of Neuroradiology, Ludwig Maximilian University, Munich, Germany

**Keywords:** Aneurysm, Endovascular, Endosaccular, Web, Coils

## Abstract

**Purpose:**

This study aims to compare endosaccular flow disruptor (EFD) for treatment of basilar tip aneurysm (BTA) with coiling in terms of safety and efficacy.

**Methods:**

We retrospectively reviewed patients treated with an EFD for BTAs at our institution between 2013 and 2019 to standard coiling from the same period (control group). Patient demographics, aneurysm characteristics, procedural data, complications and clinical and angiographic outcome were compared between groups.

**Results:**

Twenty-three (56%) patients were treated with an EFD and eighteen (44%) patients were treated with coiling. Average aneurysm size was 8 mm in the EFD group and 6.9 mm in the coiling group, respectively (*P* = 0.2). Average fluoroscopy time, treatment DAP and air kerma were 33 min, 76 Gycm^2^ and 1.7 Gy in the EFD group and 81 min, 152 Gycm^2^ and 3.8 Gy in the coiling group, respectively (*P* < 0.001). In the EFD group, clinically relevant thromboembolic complications occurred in one patient (4%) vs. in 5 patients (28%) in the coiling group (*P* = 0.07). In each group, 4 patients had an unfavourable outcome at discharge (*P* = 0.7). Adequate occlusion rates were 96% in the EFD group and 100% and coiling group. Six (26%) patients were prescribed long-term antiplatelet therapy in the EFD group vs. eleven (61%) patients in the coiling group (*P* = 0.02).

**Conclusion:**

Both treatment concepts provided similar technical success and safety. However, procedure time, radiation exposure and a need for long-term antiaggregation were lower with EFD.

## Introduction

Endovascular occlusion is the treatment of choice for basilar tip aneurysm (BTA). However, the anatomy of the basilar apex and the often broad-based configuration of BTAs may make treatment challenging.

A variety of different techniques has been developed to enable safe and successful endovascular treatment of bifurcation aneurysms including balloon and stent assistance in various configurations and recently developed neck-bridging devices [1, 2]. However, all these techniques require several steps, making the procedure complex and prone to device- and procedure-related complications.

The Woven EndoBridge (WEB; Microvention/Terumo, Aliso Viejo, CA, USA) is deployed completely endosaccular in order to reduce the inflow into the aneurysm at the level of the neck, thus leading to thrombus formation and potentially shrinkage of the aneurysm sac. The technical success and safety of WEB was supported by several studies resulting in widespread use for both ruptured and unruptured aneurysms [3–7].

Recently, the Contour device (Cerus Endovascular, Fremont, CA, USA) was introduced; similar to WEB, it is designed to disrupt the inflow into the aneurysm sac at the level of the neck of the aneurysm. The device is composed of nitinol wires forming a dual-layer mesh and has a flat, disc-like configuration when unconstrained. Once optimally deployed at the aneurysm neck, it adopts a conical shape, covering the lower part of the aneurysm and the neck [8, 9].

Several studies compared different techniques for the treatment of intracranial aneurysms [10, 11]. However, to the best of our knowledge no studies are available comparing the safety and efficacy of different endovascular techniques specifically in BTAs.

In the current study, we compared endosaccular flow disruption to conventional methods for treatment of BTAs in terms of safety and efficacy.

## Material and methods

We retrospectively reviewed consecutive patients who underwent endovascular treatment for saccular BTA at our institution between January 2013 and December 2019. Local ethics committee approved this study and requirement of patient consent was waived because of the retrospective nature of the study.

The inclusion criteria were defined as (1) successful endovascular treatment of a BTA using an endosaccular flow disruptor and (2) successful endovascular treatment of a BTA by stand-alone coiling, balloon-assisted coiling (BAC) or stent-assisted coiling (SAC).

Cases fulfilling the first inclusion criterion formed the “endosaccular flow disruptor (EFD) group” and the rest formed the “coiling group”.

Exclusion criteria were (1) recurring aneurysms and (2) aneurysms treated with an endosaccular flow disruptor using stent assistance.

### Procedure

The technique chosen for aneurysm occlusion was left to the discretion of the neurointerventionalist. Generally, stand-alone coiling or WEB was used as standard approach. In wide-necked aneurysms, anatomical features requiring stent or balloon assistance or the Contour were as follows: irregular shape, daughter sacs, low aspect ratio (≤ 1.3) or very small or large aneurysm size (i.e. not covered by the available sizes of WEB). Configuration and size of the posterior cerebral arteries was also a factor when choosing between BAC and SAC or EFD.

All procedures were performed on a biplane angiosuite (Siemens, Erlangen, Germany) under general anaesthesia. A bolus of intravenous heparin (5000 IU) was administered after groin puncture, followed by smaller doses to maintain an activated clotting time (ACT) of 2 to 3 times of the baseline. After navigating a guiding catheter into the larger vertebral artery, the aneurysm was catheterized with a dedicated microcatheter over a 0.014-in. microguidewire.

### EFD group

Endosaccular flow disruptors that have been used were WEB single layer (SL), WEB single-layer sphere (SLS) and the Contour device.

WEB was delivered through a VIA microcatheter (Microvention/Terumo, Aliso Viejo, CA, USA). The appropriate WEB size was selected to slightly exceed the aneurysm width as recommended by the manufacturer. Contour was delivered through a Headway 27 microcatheter (Microvention/Terumo, Aliso Viejo, CA, USA).

### Coiling group

Coiling was performed through a 0.017-in. SL-10 microcatheter (Stryker, Kalamazoo, MI, USA). In all BAC procedures, Sceptre balloons (Microvention/Terumo, Aliso Viejo, CA, USA) were used.

For SAC, single-stenting or Y- and T-stenting techniques were used. Stent types implanted were Solitaire AB (Covidien, Irvine, CA, USA), Neuroform EZ and Atlas (Stryker, Kalamazoo, MI, USA), LEO Baby (Balt, Montmorency, France) or LVIS Jr. (Microvention/Terumo, Aliso Viejo, CA, USA).

In one case, the eCLIPs (Endovascular Clip System; Evasc Medical Systems Corp., Vancouver, BC, Canada) device was used to reconstruct the neck.

### Antiaggregation therapy

#### Unruptured aneurysms

Patients scheduled for elective treatment with an EFD or a stent were premedicated with acetylsalicylic acid (ASA) 100 mg and clopidogrel 75 mg daily, started 5 to 7 days before treatment. In cases treated with EFD, ASA monotherapy was continued for a minimum of 4 weeks. If a stent was used, a dual antiplatelet regimen (ASA 100 mg and clopidogrel 75 mg daily) was required for 4–6 months after the procedure, followed by life-long ASA 100 mg/day.

In cases treated with stand-alone or balloon-assisted coiling, depending on the size of the neck and the protrusion of coil mass into the parent artery, ASA monotherapy was continued for a minimum of 3 months.

#### Ruptured aneurysms

In patients with ruptured aneurysms treated with SAC, tirofiban (Aggrastat; Merck, New York, USA) was administered during the procedure and usually continued for 12 h after the procedure, followed by a loading dose of ASA (500 mg) and clopidogrel (300 mg). Antiplatelet therapy was continued as described above for unruptured aneurysms.

In EFD or stand-alone coiling cases with wide neck and device protrusion into the parent vessel, short-term monotherapy with ASA was considered after securing the rupture point.

If acute thrombosis occurred during the procedure, intravenous tirofiban was started irrespective of the rupture status, usually continued for 12 h after the procedure and followed by a double antiplatelet therapy as described above.

Antiplatelet therapy ending within the first 6 months after the procedure is considered short term and therapy continuing more than 6 months is considered long-term therapy.

Drug response was tested in all patients (Multiplate® Analyser; Roche, Basel, Switzerland). An insufficient response to either drug was managed either by dose escalation or substitution with an agent such as prasugrel.

### Data collection

Patient demographics, aneurysm characteristics, procedural data, procedure-related complications and clinical and angiographic outcome at follow-up were retrospectively obtained from the medical charts. Procedural radiation exposure was measured as fluoroscopy time, air kerma (Gy) and dose area product (DAP, Gycm^2^). The Fisher scale (1: no subarachnoid (SAH) or intraventricular haemorrhage (IVH); 2: diffuse thin SAH without IVH; 3: thick SAH without IVH; 4: IVH or intracerebral haemorrhage with or without SAH) was used to evaluate the extent of SAH on CT and the WFNS (World Federation of Neurosurgical Societies) grading system (1: GCS (Glasgow Coma Scale) 15 without deficit; 2: GCS 13–14 without deficit; 3: GCS 13–14 with focal neurological deficit; 4: GCS 7–12 with or without deficit; 5: GCS < 7 with or without deficit) was used to evaluate the clinical status. Wide neck was defined either as a neck >4 mm or an aspect ratio < 1.6. Clinical outcome was evaluated by the modified Rankin Scale (mRS) before treatment and at discharge. Unfavourable outcome was defined as mRS > 2.

### Angiographic control and retreatment

Our follow-up protocol consists of angiographic controls at 6 and 24 months after the procedure using DSA (digital subtraction angiography) in the majority of cases and magnetic resonance angiography (MRA) or computed tomography angiography (CTA) in other cases. The Raymond-Roy occlusion classification was used to assess aneurysm occlusion at follow-up (grade I: complete occlusion; grade II: neck remnant; grade III: aneurysm remnant). Complete occlusion and neck remnants were defined as adequate occlusion.

### Statistical analysis

Continuous variables were presented as means, percentages and ranges. They were tested for normality using the Shapiro-Wilk test and compared between the EFD group and coiling group using the 2-sided unpaired Student *t* test (for normally distributed data) and the Mann-Whitney *U* test (for non-normally distributed data).

Categorical variables were expressed as names or numbers with percentages and compared between the groups using the *χ*^2^ and the Fisher exact test, when appropriate.

Univariate logistic regression analysis was performed to test the predictive power of treatment type on short- and long-term antiplatelet use and angiographic and clinical outcome. A linear regression analysis was made to analyse the relationship between aneurysm size, height, neck width or aspect ratio and the amount of fluoroscopy time or radiation exposure. All calculations were performed using SPSS software Version 25.0 (IBM, Armonk, New York, USA). A *P* value <0.05 was considered statistically significant.

## Results

### Patient and aneurysm characteristics

Forty-one patients who underwent endovascular treatment for a saccular BTA between 2013 and 2019 were included. Twenty-three (56%) patients were treated with an EFD and eighteen (44%) patients were treated with other techniques. The characteristics of patients and aneurysms from each group are shown in Table 1. All SAHs were grade 4 according to the Fisher scale. The median WFNS score of SAH patients was 4.

**Table 1:** Characteristics of patients, aneurysms and procedures and list of complications and follow-up

	EFD (*n* = 23)	Coiling (*n* = 18)	*P* values
Mean age (range)	61 (35–81)	58 (40–76)	0.5
Male sex	8 (35%)	6 (33%)	0.9
Ruptured aneurysms	4 (17%)	9 (50%)	0.02
Mean size in mm (range)	8 (3.1–16)	6.9 (3.1–12)	0.2
Mean neck size in mm (range)	4.4 (2.3–6.8)	3.9 (2.1–6.7)	0.1
Wide-necked aneurysms	20 (87%)	12 (67%)	0.1
Mean aspect ratio (range)	1.4 (0.9–2.8)	1.5 (0.7–2.8)	0.7
Mean fluoroscopy time in min (range)	33 (8–108)	81 (30–143)	< 0.001
Mean dose area product in Gycm^2^ (range)	76 (11–199)	152 (58–298)	< 0.001
Mean air kerma in Gy (range)	1.7 (0.3–5)	3.8 (1.5–7.8)	< 0.001
Thromboembolic complications	5 (21%)	6 (33%)	0.5
Clinically relevant thromboembolic complications	1 (4%)	3 (17%)	0.3
Unfavourable clinical outcome	4 (17%)	4 (22%)	0.7
Adequate aneurysm occlusion	22 (96%)	18 (100%)	1
Retreatment	1 (4%)	0 (0%)	1
Short-term antiplatelet medication	16 (70%)	12 (67%)	0.8
Long-term antiplatelet medication	6 (26%)	11 (61%)	0.02

### Aneurysm treatment and procedural radiation exposure

All but two cases were performed via transfemoral approach. In two cases (one in each group), transbrachial approach was selected because of chronic bilateral iliac artery occlusions. Endovascular treatment techniques are presented in Table 2.

**Table 2:** Endovascular treatment techniques

Treatment group	Treatment method	Number of patients
EFD	WEB	18 (78%)
	Contour	4 (17%)
	WEB + coils	1 (5%)
Coiling	Coiling	7 (39%)
	BAC	4 (22%)
	SAC	6 (33%)
	Neck-bridging device	1 (6%)

In three SAH patients (17%) from the coiling group, endovascular treatment of an aneurysm at another location (two anterior communicating artery and one superior cerebellar artery (SCA) aneurysms) was necessary during the same session as the BTA. All of them were treated with coils. In one SAH patient, BTA and the additional SCA aneurysm were covered with the same stent and occluded with coils.

Procedural radiation doses and procedure times are reported in Table 1. In the coiling group, after exclusion of the patients with other aneurysms treated during the same session, the average fluoroscopy time, DAP and air kerma dropped to 76 min, 145 Gycm^2^ and 3.7 Gy, respectively, maintaining the significant difference from the EFD group.

According to linear regression analysis, there was no significant relationship between aneurysm size, height, neck width or aspect ratio and the amount of fluoroscopy time or radiation exposure.

### Complications

In the EFD group, adverse events occurred in five WEB patients (21%). All of them were thromboembolic events and happened in elective cases. However, only one of the patients had a transient neurologic deficit and the MR Imaging showed multiple embolic lesions in multiple vascular territories. Others were clinically silent.

In the coiling group, complications occurred in seven patients (39%). Six (33%) of them were thromboembolic events (5 in SAH patients). In only 3 (17%) patients thromboembolic complications were clinically relevant. One of them presented itself 4 months after the intervention after cessation of clopidogrel. All others occurred were periprocedural. In one elective case, a LEO Baby 3/25 stent was positioned at the level of the aneurysm neck initially but dislocated during deployment. It was then safely placed in the distal cervical segment of the vertebral artery. The aneurysm was subsequently coiled with stent assistance using two LVIS Jr. stents in T-configuration. The patient showed no postoperative neurological deficit.

There was no statistical significance between two groups regarding the thromboembolic complication rates (*P* = 0.5).

Including only clinically relevant thromboembolic complications, the rates dropped down to 4% (1/23) and 17% (3/18) in the EFD group and coiling group, respectively. The difference was still not statistically significant (*P* = 0.3).

No haemorrhagic event was recorded.

### Clinical outcome

In each group, 4 patients (17% in the EFD group and 22% in the coiling group) had an unfavourable outcome at discharge (*P* = 0.7). From the four patients, two had SAH in the EFD group and all had SAH in the coiling group.

In the EFD group, two of the SAH patients (9%) died; one due to multiple cerebral infarctions because of intractable cerebral vasospasm and the other one due to multi-organ failure. Remaining cases with unfavourable outcome had already high mRS scores because of pre-existing comorbidities.

In the coiling group, one patient (5%) presented with SAH and treated with SAC died because of haemorrhagic transformation of a large posterior cerebral artery infarction due to stent occlusion. Other patients had unfavourable scores because of SAH-related incidences.

### Angiographic outcome

Median follow-up was 8 months (min 1, max 60). Eight patients (5 in the EFD group and 3 in the coiling group) were lost to follow-up. Sixteen patients had no DSA, but MR or CT angiography at follow-up. In the EFD group, adequate occlusion rate was 96% (19 complete occlusions, 3 neck remnants and 1 aneurysm remnant). Only one patient (4%) who was initially treated with a WEB device showed recurrence after 11 months and was retreated with BAC. In the coiling group, adequate occlusion was obtained in 100% of the patients (16 complete occlusions and 2 neck remnants). Adequate occlusion rates were not significantly different between the two groups (*P* = 1).

According to univariate logistic regression analysis, treatment type was not predictive of angiographic and clinical outcome.

### Antiplatelet medication

Antiaggregation status of patients at baseline, < 6 months and > 6 months after the procedure is presented in Table 3. In short term, 16 (70%) patients in the EFD group took single or double antiplatelets vs. 12 (67%) patients in the coiling group (*P* = 0.8). Six (26%) patients were prescribed long-term antiplatelet therapy in the EFD group vs. eleven (61%) patients in the coiling group (*P* = 0.02). Two patients in the EFD group took double antiplatelet medication for 6 months after the procedure (9%) vs. eight (44%) patients in the coiling group (*P* = 0.01). However, treatment type was not a predictor of short- or long-term antiplatelet therapy.

**Table 3:** Antiplatelet regimen at baseline, < 6 months and > 6 months after the procedure

Number of antiplatelets	EFD group			Coiling group		
		Treatment duration			Treatment duration	
	Baseline	< 6 months	> 6 months	Baseline	< 6 months	> 6 months
0	19 (83%)	7 (30%)	17 (74%)	18 (100%)	6 (33%)	7 (39%)
1	4 (17%)	14 (61%)	6 (26%)	0 (0%)	4 (22%)	10 (55%)
2	0 (0%)	2 (9%)	0 (0%)	0 (0%)	8 (45%)	1 (6%)

## Discussion

In the current study, we compared endosaccular flow disruption with conventional methods (stand-alone coiling, BAC and SAC) for treatment of BTAs in terms of safety and efficacy. We did not find a significant difference between the treatment groups regarding the thromboembolic complication rate, as well as the clinical and final angiographic outcome. However, procedural fluoroscopy time and radiation exposure were significantly lower in patients who were treated with endosaccular flow disruptor devices. In addition, they were significantly less likely to have an indication to take antiplatelets in long term.

BTAs are special among cerebral aneurysms in several respects: surgical treatment is usually not an option, but due to the often broad-necked configuration (up to 60% according to Lozier et al.), pure coiling is also in many cases not possible [12–16].

Rates of recurrence after stand-alone coiling have been reported to be as high as 25 to 30% in large and wide-necked aneurysms [17, 18]. While additional devices such as balloons, self-expanding stents or neck-bridging devices may improve the coil packing density and thereby the efficacy of endovascular coil embolization on one hand, they increase the complexity of the procedure on the other hand, which in turn may increase the risk for complications [19–21]. Furthermore, antiplatelet medication is mandatory for all patients after any stent treatment, which may complicate subsequent surgical procedures, especially for patients after SAH [21, 22].

The WEB and Contour devices represent a completely different concept: different from stents and intraluminal flow diverter stents, these dense-meshed devices are placed completely in the aneurysm sac and aim to disrupt the blood flow entering and exiting the sac at the level of the neck in order to promote stagnation and thrombus formation. The WEB device proved efficacy and safety in multiple prospective and retrospective multi-centred studies, especially in the treatment of ruptured and wide-necked aneurysms [3–7].

Currently, there are two studies that compare stand-alone coiling and WEB treatment, as well as SAC and WEB [10, 11]. Kabbasch et al. included aneurysms at all anatomical locations in the evaluation. They reported a significantly higher complication rate in the SAC group (14/66 aneurysms, 21%) compared with the WEB group (8/66 aneurysms, 12%). The complete occlusion rate was higher in aneurysms treated with the WEB device (41/47, 87%) compared with aneurysms treated by coiling (31/51, 61%) and similar to those treated by SAC (55/66, 84% vs. 56/66, 85%). Retreatment rates were significantly higher in the coiling group (9/51, 18% vs. 2/47, 4%) and similar between the WEB (7/66, 11%) and the SAC (8/66, 12%) group.

Other potential advantages of the EFDs, such as potential reduction of procedural fluoroscopy time and radiation dose have, however, not been addressed yet.

### Complications

The rate of clinically relevant thromboembolic events was higher in the coiling group (17%) than in the EFD group (4%), however without statistical significance (*P* = 0.3). The thromboembolic complication rate in the EFD group was in compliance with the literature [3–7].

The coiling group showed a higher rate than previously reported. In large coiling series including only BTAs, thromboembolic complication rates varied from 6 to 12% [12–14, 16, 17]. Explanations might be small size of our study group and significantly higher numbers of ruptured cases in the coiling group.

### Radiation exposure

Radiation exposure in endovascular aneurysm therapy has so far received little attention. Differences in individual aneurysm geometry and vessel anatomy, as well as in experience levels and treatment preferences of neurointerventionalists, make a comparison of radiation exposure and a definition of acceptable thresholds difficult.

Direct measurement of the absolute entrance skin doses is not practicable and therefore surrogate markers such as DAP (Gycm^2^) and air kerma (Gy) are usually used [23–27].

The threshold for deterministic skin damage such as erythema or hair loss is considered to be at 2 to 3 Gy [27, 28]. Struelens et al. proposed a DAP trigger level of 220–330 Gycm^2^ as a threshold for skin effects in cerebral embolization procedures [26].

In a study of Peterson et al. [23] including 702 neurointerventional procedures, skin entrance doses exceeding 2 Gy occurred in 73% of procedures. After almost 40% of them, patients reported changes of their skin or hair and 30% of the changes were permanent. Increasing skin dose was significantly associated with permanent hair loss.

Ihn et al. evaluated 371 aneurysm treatments nationwide in 2015 [25]. Total mean DAP, air kerma and fluoroscopy time were 219 Gycm^2^, 3.3 Gy and 51 min, respectively. The reported radiation exposure exceeded the cited threshold values but the patients were not specifically followed up for radiation-induced skin or hair changes.

Data regarding the radiation exposure in treatment of intracranial aneurysms with the WEB device is available from a prospective multi-centre study including 150 aneurysms. Arthur et al. reported an average procedural fluoroscopy time of 30 min and an average air kerma of 2.7 Gy [7]. Our findings in the EFD group (33 min, 76 Gycm^2^, 1.7 Gy) were comparable with those figures. Both the procedural time and the radiation dose were higher in the coiling group (81 min, 152 Gycm^2^, 3.8 Gy); however, these numbers are still in compliance with the literature [24, 25].

Technical simplicity is certainly the major reason for faster procedure times and low radiation exposure in treatments with EFD. SAC, for example, requires multiple steps including catheterization of the distal parent vessel, stent deployment, catheterization of the aneurysm sac and filling it with multiple coils. Usually several control injections are needed. In EFDs, in particular for WEB, exact measurements and device selection prior to the procedure are necessary, but the procedure itself after catheterization consists only of device deployment.

### Angiographic outcome

In our study group, both treatment groups had similar rates of adequate occlusion (22/23, 96% in the EFD group vs. 18/18, 100% in the coiling group). Retreatment was necessary only in one patient after WEB and in none of the aneurysms treated with other techniques.

In general, complete occlusion seems to be less frequently achieved in BTAs and retreatment is more often necessary when compared with other aneurysm localizations [14, 17, 29, 30]. In a systematic review including 226 coiled BTAs, Lozier et al. [16] reported an initial complete or near-complete aneurysm occlusion in 88% of the patients; however, the recanalization rate was 26% with a 0.7% annual risk for recurrent haemorrhage. In another study by Henkes et al. [12] including coil embolization of 316 BTAs, recurrence, retreatment and recurrent haemorrhage rates were found to be as high as 35%, 15% and 5%, respectively. After the initial embolization procedure, a 90 to 100% occlusion rate was achieved in 86% of the aneurysms.

In the cumulative population of three prospective WEB studies, Pierot et al. observed an adequate occlusion rate of 81% and retreatment rate of 9% at 2-year follow-up [4]. Just as in our study, Kabbasch et al. found equal adequate occlusion rates between WEB and SAC (94%) in their series including 66 wide-necked bifurcation aneurysms from each group [11].

### Antiplatelet medication

So far, there has been no standard regarding the use of antiplatelet medication after endovascular aneurysm treatment. This applies to stand-alone coiling, as well as to SAC and EFD. The peri- and postprocedural antiplatelet regimen is usually left to the neurointerventionalists’ discretion. In cases treated without stent assistance, usually a short-term single or double antiplatelet therapy is given, especially in wide-necked aneurysms or when the parent artery or incorporated vessels are compromised by the implant. In patients treated by stent-assisted coiling, a short-term double antiplatelet therapy is required.

In the multi-centred prospective WEB studies, antiplatelet therapy was not mandatory. However, the majority of the patients received double antiplatelet medication prior and during the procedure (69% in the WEB-IT study and 45% in the WEBCAST and French Observatory group) [4, 7].

In the WEB-IT study, 74% of patients had short-term antiplatelet therapy in keeping with our results (70%) from the EFD group. In the coiling group, the overall rate was similar (67%), but the percentage of patients using double antiplatelets was higher (67% in the coiling vs. 12% in the EFD group) [7].

In long term, patients in the coiling group (61%) were significantly more often prescribed antiplatelets than in the EFD group (26%). As a comparison, that rate was 48% in the WEB-IT study group [7]. Unfortunately, we could not find any comparable study reporting antiaggregation use in coiled aneurysms.

### Limitations

Major drawbacks of this study are mainly its retrospective, single-centred and non-randomized nature and the relatively small number of included cases. The method chosen to occlude the aneurysms was based on the discretion of the neurointerventionalist and various factors such as the surgeon’s experience, rupture status or patients’ comorbidities may have influenced these decisions. Thus, we cannot exclude a potential selection bias, because some of the aneurysm in the coiling group may have had an unfavourable configuration for EFD. Furthermore, heterogeneity of the occlusion techniques used (stand-alone, balloon- or stent-assisted coiling) and larger proportion of ruptured aneurysms among the coiling group might confound a meaningful comparison between two main treatment strategies.

Only one patient was included who was treated with a dedicated bifurcation stent (eCLIPs) and our data thus does not allow any conclusion about treatment with these devices.

## Conclusion

Our data provides the first comparative analysis of endosaccular flow disruption with other treatment methods for endovascular management of basilar tip aneurysms.

Both treatment concepts provided similar technical success and safety. However, procedural time, radiation exposure and the indication for long-term antiaggregation were significantly in favour of EFD. It may thus be argued, that with both strategies deemed suitable, EFD should be preferred.
